# Ushering the Cactvs Toolkit into the Python Age (without breaking the legacy)

**DOI:** 10.1186/1758-2946-6-S1-P34

**Published:** 2014-03-11

**Authors:** Wolf D Ihlenfeldt

**Affiliations:** 1 Xemistry GmbH, Königstein, D-61462, Germany

## 

The Cactvs Chemoinformatics Toolkit is probably the most powerful general-purpose chemical information processing toolkit on the market. Since its inception about twenty years ago, its main language for rapid script development has been Tcl – at that time a language at the forefront of lazily-typed rapid prototyping and interface programming language design.

While Tcl is still actively maintained, and does provide features not matched by many of the nowadays more popular competitors – most notably impressive multi-threading capabilities, which are fully accessible from within the toolkit – history has passed on. Tcl has undeniably fallen out of the public eye, and there is an understandable reluctance by users to learn new languages which are effectively only used by one of their tools.

This problem has finally been addressed. The Cactvs toolkit is now available with Python as a second alternative (or parallel) interface language. The new interface closely follows the established Tcl command patterns to support easy migration by experienced users, while still providing true “pythonesque” constructs. Since significant functionality of the toolkit is implemented as external Tcl script function snippets, and future enhancements will probably preferably be coded in Python without providing also a Tcl port, providing automatic and fully transparent access to language-mismatched components has been an important and rather peculiar design goal.

Examples of the new toolkit scripting capabilities shall be presented, as well as a documentation of the challenges involved in the design of a parallel multi-language interface to a large software system.

